# Microfluidics in High-Throughput Drug Screening: Organ-on-a-Chip and *C. elegans*-Based Innovations

**DOI:** 10.3390/bios14010055

**Published:** 2024-01-21

**Authors:** Sunhee Yoon, Dilara Kilicarslan You, Uiechan Jeong, Mina Lee, Eunhye Kim, Tae-Joon Jeon, Sun Min Kim

**Affiliations:** 1 Department of Biological Sciences and Bioengineering, Inha University, Incheon 22212, Republic of Korea; yoonsh0912@inha.ac.kr (S.Y.); dilarakilicarslanscience@gmail.com (D.K.Y.); dlalsdkmina1114@gmail.com (M.L.); dsm5477@gmail.com (E.K.); 2 Department of Mechanical Engineering, Inha University, Incheon 22212, Republic of Korea; 3 Department of Biological Engineering, Inha University, Incheon 22212, Republic of Korea; 4 Biohybrid Systems Research Center (BSRC), Inha University, Incheon 22212, Republic of Korea

**Keywords:** biochips, *C. elegans*, drug screening, microfluidics, cell chip, organ-on-a-chip

## Abstract

The development of therapeutic interventions for diseases necessitates a crucial step known as drug screening, wherein potential substances with medicinal properties are rigorously evaluated. This process has undergone a transformative evolution, driven by the imperative need for more efficient, rapid, and high-throughput screening platforms. Among these, microfluidic systems have emerged as the epitome of efficiency, enabling the screening of drug candidates with unprecedented speed and minimal sample consumption. This review paper explores the cutting-edge landscape of microfluidic-based drug screening platforms, with a specific emphasis on two pioneering approaches: organ-on-a-chip and *C. elegans*-based chips. Organ-on-a-chip technology harnesses human-derived cells to recreate the physiological functions of human organs, offering an invaluable tool for assessing drug efficacy and toxicity. In parallel, *C. elegans*-based chips, boasting up to 60% genetic homology with humans and a remarkable affinity for microfluidic systems, have proven to be robust models for drug screening. Our comprehensive review endeavors to provide readers with a profound understanding of the fundamental principles, advantages, and challenges associated with these innovative drug screening platforms. We delve into the latest breakthroughs and practical applications in this burgeoning field, illuminating the pivotal role these platforms play in expediting drug discovery and development. Furthermore, we engage in a forward-looking discussion to delineate the future directions and untapped potential inherent in these transformative technologies. Through this review, we aim to contribute to the collective knowledge base in the realm of drug screening, providing valuable insights to researchers, clinicians, and stakeholders alike. We invite readers to embark on a journey into the realm of microfluidic-based drug screening platforms, fostering a deeper appreciation for their significance and promising avenues yet to be explored.

## 1. Introduction

Drug screening, carried out during the drug discovery process, typically refers to the process of identifying potential therapeutic compounds or natural products that have a desired effect on a target biological system by modulating the activity of a specific biomolecule [1,2]. This is the process of evaluating the pharmacological activity and toxicity of potential drug candidates and is a critical step in the drug discovery process.

Conventional drug screening platforms mainly consist of in vitro (cell-based) assays and in vivo (animal-based) models. The predominant in vitro method is high-throughput screening (HTS) using cell lines in multi-well plates [3,4]. In vitro assays typically use immortalized cell lines to test the effects of different drug candidates on specific targets or pathways. These assays measure changes in cellular activity in response to the drug, which can include changes in the expression of specific proteins, cell proliferation, or cell death. These assays can be performed at high-throughput using robotics, liquid handling devices, and automated microscopy. In vivo models, on the other hand, use animal models such as mice to evaluate the efficacy and toxicity of potential drug candidates at the whole organism level [5,6]. Conventional methods are commonly employed, yet present considerable drawbacks. Primarily, cost is a major concern. Specifically, while HTS yields rapid high-throughput screening, it demands intricate and costly analytical systems, in addition to the significant quantity of samples required, thereby constituting a substantial impediment to drug screening perpetuated by this method [7,8,9]. Conventional cell-based assays often use single cell layers that lack the structural and functional complexity of human tissues. This constraint hinders accurate drug response predictions in complex biological systems [10,11]. In addition, the use of animals in research raises ethical considerations, and there is an ongoing societal push to reduce, refine, and replace the use of animals in research [12]. Finally, in addition to the in vitro models mentioned above, in vivo models can also be costly and time-consuming to develop new drugs based on, due to the value of the animals themselves, the cost of maintaining them and the length of time required to obtain data [13].

As an alternative to conventional drug screening platforms, microfluidic systems are emerging as leaders in technology to revolutionize drug discovery processes [14]. Microfluidics, rooted in the fields of engineering and physics, is a multidisciplinary approach that started being applied to biotechnology in the late 1990s, based on suitable fabrication materials and techniques [15]. Since the early 2000s, microfluidic systems have been increasingly applied across a diverse range of biotechnology applications, with notable momentum in the field of drug screening [16]. Microfluidic systems refer to a system capable of manipulating and controlling fluids within tens to hundreds of micrometer-sized channels, typically in the picoliter to microliter range. The system’s ability to manipulate fluids, coupled with the ability to handle small amounts of reagents, provides a high level of control and precision, making it an ideal system for high-throughput screening (HTS) and drug discovery.

Notably, microfluidic systems can be designed and fabricated as intended by the researcher to maximize their effectiveness. Based on these user-friendly characteristics, microfluidic systems utilized as various research platforms can be extended to cell/organ chips and organism-level chips for drug screening by combining human-derived cells and the model organism such as *C. elegans*, respectively.

Cell/organ chips based on microfluidic systems provide advantages such as 3D cell culture, co-culture, and the in vitro replication of the microenvironment of living tissues and organs by closely mimicking in vivo conditions, resulting in more accurate and physiologically relevant results than traditional 2D cell culture models [16]. Furthermore, microfluidic devices enable immediate real-time monitoring and manipulation of the cellular microenvironments and can also reduce costs through smaller sample sizes. Cell/organ chips typically consist of a network of channels and chambers with living cells, allowing researchers to create dynamic 3D models of tissues and organs in vitro. The primary focal point of this platform is the implementation of dynamic 3D models. Unlike more conventional cell-based platforms, microfluidic systems have the capability to connect to pumps and simulate actual blood circulation [17,18]. Consequently, these microchips can be customized to replicate numerous physiological conditions such as fluid shear stresses, mechanical strains, and chemical gradients, which are imperative in ensuring the optimal function of living tissue. The utilization of cell or organ chips in pharmaceutical screening can consequently result in more precise and dependable testing of promising drug candidates. For example, Ewing’s sarcoma (ES) mimetic organ-on-a-chip was proposed to overcome the limitations of conventional drug screening models that do not closely reproduce human physiology [19]. In this study, a drug (Linsitinib) that had shown good efficacy against the target disease in preclinical studies, but showed significantly lower efficacy in clinical studies, was retested by building an organ-on-a-chip. When administered at the same dose as in the previous clinical test, the three-dimensional ES model produced results much closer to the response observed in the clinical trial. As such, cell/organ chips based on microfluidic systems are an optimal platform to closely simulate the in vivo environment, effectively overcoming the limitations of existing platforms and enabling more accurate testing of potential drug candidates.

*C. elegans* is a tiny organism, measuring only 1 mm. It has a well-differentiated nervous system and up to 60% genetic homology with humans, making it a popular model organism for a variety of research applications [20,21,22,23,24]. *C. elegans* offers numerous advantages, particularly as a drug screening platform. Primarily, *C. elegans* was the first multicellular organism to have its entire genome sequenced in 1998 [25], and there is an institution (Caenorhabditis Genetic Center (CGC), Minneapolis, MN, USA) that maintains a significant number of mutants separately. These are allowing researchers to perform gene-level analysis with relative ease and access to a wide variety of mutants. This is a highly effective and valuable model for identifying potential drug targets and studying drug mechanisms of action. In addition, *C. elegans* is easy to culture and maintain in the laboratory compared to other in vivo models, and has a relatively short lifespan and generation cycle compared to other model organisms. Furthermore, its advantages include the lack of ethical issues. A significant advantage of *C. elegans* as a drug screening platform is the ability to assess drug efficacy/toxicity at the organism level, and the fact that drugs that work in *C. elegans* are likely to work in humans. Its status as a prominent drug screening platform is largely due to these advantageous features.

However, because of its diminutive size, with a maximum length of only 1 mm, *C. elegans* is out of scale to the conventional platforms of several centimeters that are commonly used, resulting in low research efficiency. In this point, microfluidic systems with a scale of tens to hundreds of micrometers can not only provide adequate experimental and culture space considering the size of *C. elegans* (maximum length of 1 mm, maximum body width < 100 μm), but also allow researchers to design the structure as intended, thus providing researchers with an optimal *C. elegans* experimentation and research platform [26,27,28]. Typically, *C. elegans* chips consist of a network of channels and chambers used to house worms and control their environment, and researchers are using them to study the effects of various drug candidates on development, behavior, aging, etc., at the organismal level [29,30,31,32,33]. For example, a microfluidic-based system was developed to effectively chamber and culture a single *C. elegans* with the goal of building an organism-level drug screening platform. In this system, a channel capable of accurately capturing hundreds of eggs laid by the mother could be constructed in succession, allowing high-resolution observation of the embryos until hatching [31]. A small molecule inhibitor (actin-polymerization inhibitor Cytochalasin-D (CD)) was treated in this system and its efficacy was confirmed. As such, the nematode and microfluidic system-based drug screening platform is an optimal research platform that can effectively perform organismal-level studies that cannot be performed in conventional cell-based drug screening platforms, as well as generation replacement-based studies that are almost impossible to perform in mammalian-based animal experiments.

**Figure 1:**
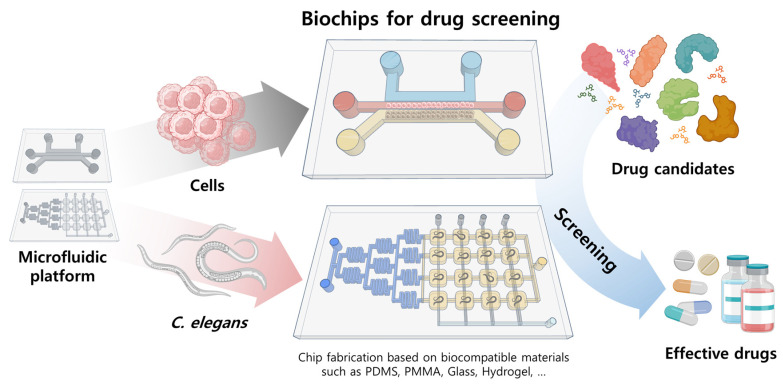
Biochips for drug screening based on cells and model organism (*C. elegans*). Created by Biorender.com (BioRender.io, Toronto, ON, Canada) and Tinkercad.com (Autodesk, Inc., San Rafael, CA, USA). (Both websites accessed on 24 November 2023).

This review focuses on drug screening applications using cell/organ chips and *C. elegans*-based biochips (*C. elegans* chip) (Figure 1). We provide a brief overview of fundamental microfluidic systems employed as the foundation for drug screening. The subsequent sections examine the cell/organ chip and *C. elegans* chip, respectively, and analyze their specificities as drug screening platforms, along with case studies evaluating their efficacy. Finally, the accomplishments of drug screening platforms based on microfluidic systems are summarized up to the present time, and future applications are discussed.

## 2. Overview of Microfluidic Systems Utilized as Drug Screening Systems and How They Are Fabricated

Microfluidic systems gained spotlight in biological applications in the 1990s with the development of soft lithography technology [34]. Microfluidic systems are systems with dimensions of tens to hundreds of micrometers that handle very small volumes of fluid, ranging from 10^−18^ to 10^−9^ L. Due to the considerable differences in dimensions and volume in comparison with the conventional systems, and the resulting drastic reduction in the amount of sample used in various experiments, this system has begun to attract attention as a new, next-generation system with low cost and maximized efficiency [35]. In addition, based on its small size, microfluidic systems were easy to combine with various equipment to control the flow of internal fluids in a precise manner, which significantly improved the accuracy and precision of experiments. Based on these advantages, various studies have been carried out in the 2000s to apply microfluidic systems to biology [36,37].

Microfluidic systems utilized as drug screening platforms must, among other things, be composed of biocompatible materials. The aim of a drug screening platform is to evaluate the efficacy or toxicity of candidate compounds for the treatment of a disease. Therefore, if the components of the platform exhibit cytotoxic effects, cultivating cells and model organisms in the platform becomes challenging. Hence, it is crucial to construct the system with materials that have been shown to be non-cytotoxic. Microfluidic systems used for drug screening are typically composed of materials, which has been proven to be biocompatible such as polydimethylsiloxane (PDMS) [30,31,38,39,40], and poly (methyl methacrylate) (PMMA) [41], polycarbonate (PC) [42], polystyrene (PS) [43], perfluorinated polyether (PFPE) [44], glass [45], hydrogel [46], and others.

Notably, PDMS is one of the materials that has made a significant contribution to the wider use of microfluidic systems. It was first shown in 1998 that PDMS could be utilized in the fabrication of microfluidic systems [34], and its various material properties, including biocompatibility, made it well-suited for biological applications. Primarily, PDMS has high permeability to gases such as oxygen and carbon dioxide [47], creating a suitable environment for cells to grow in. PDMS is a minimally cytotoxic material, rendering it favorable for use as a cell culture platform. In addition, its transparent nature allows for clear observation of the sample through a microscope, making it the ideal material for the fabrication of cell/organ chips. These advantages also apply to *C. elegans*-based chips, and most microfluidic systems have been designed and fabricated using PDMS. Its low fabrication cost and ease of fabrication make PDMS ideal for research utilizing microfluidic systems that require a variety of designs. The process of fabricating PDMS-based microfluidic systems is called soft-lithography [48]. A design suitable for the research purpose is first created using CAD software, and then a mold is fabricated based on the design using photo-lithography technology, typically using a photoresist [49]. PDMS replicas are then produced from the mold. The final microfluidic chip is then fabricated by forming inlets and outlets as needed and bonding them with slide glass or PDMS membranes.

PDMS has been widely used as a biochip material due to its great advantages, but it also has significant drawbacks that must be overcome in order to be used as a drug screening platform. Specifically, PDMS is inherently quite hydrophobic, making it difficult for fluids to flow through, as well as having absorption properties for small molecules (such as some drugs or other organic compounds) [50]. This is a major limitation in systems that require accurate dosing, such as drug screening [51]. Several surface modification techniques have been developed to overcome this absorption problem. Techniques such as plasma treatment or substances coating such as polyethylene glycol (PEG) can be used to increase the hydrophilicity of the PDMS surface to reduce small molecule absorption [52,53]. Another method is to saturate the surface by treating the PDMS channel with an analyte or buffer prior to the actual experiment. For example, pretreating the channel surface with 75% alcohol and a substance such as Triton X100 can effectively reduce the absorbency [54]. Importantly, this treatment also prevents protein coating on the PDMS surface, which can help to further improve the accuracy of the experiment [55]. In some cases, in addition to these surface modifications, low-temperature curing of PDMS replicas can reduce the number of uncrosslinked free oligomers, which can reduce water absorption [56].

PMMA is a type of plastic that is also used in well plates and Petri dishes used for cell culture. PMMA is non-cytotoxic, inexpensive, and, like PDMS, transparent, allowing for real-time observation of the chip inside under a microscope [57]. However, in the case of PMMA, chips cannot be fabricated in the same way as soft-lithography, but are generally fabricated by processing PMMA into the desired shape by injection molding [58], laser cutting [59], milling [60], and engraving [61].

In addition to these two materials, the recent rise of 3D printing has led to the emergence of 3D-printed biochips using a variety of non-toxic resins [62].

As a consequence, microfluidic systems utilized as drug screening platforms have been built with a variety of biocompatible materials and developed in various ways to overcome the limitations of these materials for accurate drug efficacy and toxicity testing. In the following chapters, we will take a closer look at specific examples utilized in cell/organ chips and *C. elegans* chips.

## 3. Drug Screening Applications of Various Cell/Organ and Model Organism-Based Biochips

### 3.1. Cell/Organ Chips for Drug Screening

Cell/organ chips recreate the microscopic environment of real human tissues, allowing the analysis of interactions between different cell types, and providing information on how drugs behave in vivo. This detailed mimicry helps to accurately assess the efficacy and toxicity of drug candidates.

Recent studies have shown a variety of ways to utilize these chips for various drug screening applications [63,64,65,66,67].

A cell/organ chip for drug screening purposes typically consists of a culture chamber for implementing cells/organs and microfluidic channels for handling culture media and test drugs, with additional elements such as temperature controllers and concentration gradient channels added as needed to increase its utility.

Compared to conventional 2D cell culture platforms (including drug screening platforms), the most distinctive feature of microfluidic chip-based drug screening platforms is compartmentalization through the configuration of independent chambers mentioned above. This is a major contributor to simulating the actual cellular composition as well as the 3D structure of the tissue or organ to be simulated, while ensuring the independence of each tissue/organ and simulating the interactions between tissues/organs. The structures that researchers can use to achieve this compartmentalization can be broadly divided into horizontal and vertical structures. Horizontal structures are those in which the cell culture chambers and fluid channels are located on a single plane, while vertical structures are those in which the chambers and channels are layered on different planes (Table 1).

**Figure 2:**
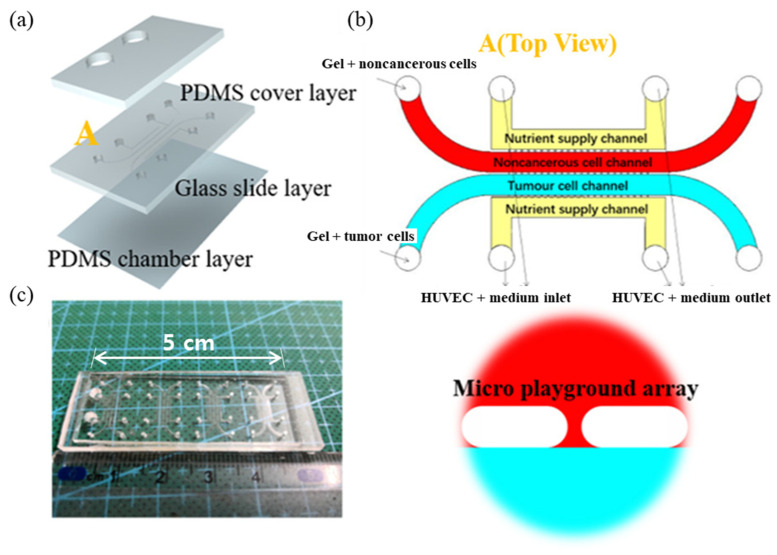
Three-dimensional tumor–macrophage system. (**a**) Illustration of the microfluidic device for coculture, (**b**) cell culture chamber layer and close-up view of the micro layer with a close-up view of the micro playground array section. (**c**) Photograph of the microfluidic system. A total four sets of tumor–macrophage systems were implemented on a single chip, and the overall horizontal length of the system was 5 cm. Reprinted with permission from [68].

Horizontal structures generally do not require complex multi-layer patterning or bonding processes, making them relatively easy to fabricate. They are also relatively free from issues such as media leakage because they do not combine multiple layers to form a chip. Since cells are located in a single layer, they are easy to observe under a microscope in real-time, and flow control is relatively easy. When co-culturing two or more cell types in a horizontal structure, rather than separating the cell culture chambers with a porous membrane, a post structure is often adopted, where a series of small posts, like dotted lines, are placed between the chambers. This allows cells to be seeded together with extra-cellular matrix (ECM) to prevent them from mixing without physically closing off each chamber, allowing them to interact with each other through the gaps between the posts. This has the advantage of simulating a more stable 3D structure while still allowing for interaction. In other cases, interactions between cell culture chambers have been achieved by connecting them with smaller micro/nanoscale channels. These are more difficult to fabricate, but have the advantage of allowing for more defined compartments and interactions. A typical example is a drug screening platform study that used a post structure to co-culture three different types of cells in a single layer, and then verified the effectiveness of the anticancer drug paclitaxel (Figure 2). In this study, a drug screening platform with a total of four channels was constructed, two of which were cell culture chambers and the other two were cell culture media supply channels [68]. In detail, two adjacent cell culture chambers are compartmentalized around a rectangular structure with rounded corners called a “micro playground”, which consists of a rectangle of 140 μm length and 90 μm width, and two semicircles that are 90 μm in diameter, spaced 30 μm apart. This micro playground is called a post, which, when a highly viscous liquid is injected into the channel at a relatively high velocity, does not leak into adjacent channels due to the surface tension of the liquid. At the same time, they allowed the cells in the channels to interact with the cell culture medium in the cell culture feed channel through the gaps and through the parts connected to each channel. The authors established a tumor–macrophage system by seeding breast cancer cells (MDA-MB-231) and three types of macrophages (U937, TAM, and MCF-10A) into the cell culture chamber along with collagen. In this system, they verified the concentration-specific efficacy of an anticancer drug (paclitaxel) according to the interaction between each macrophage and breast cancer cells, and found that even a small concentration difference showed a clear difference in drug efficacy, indicating that the platform can be utilized as a drug screening platform.

**Figure 3:**
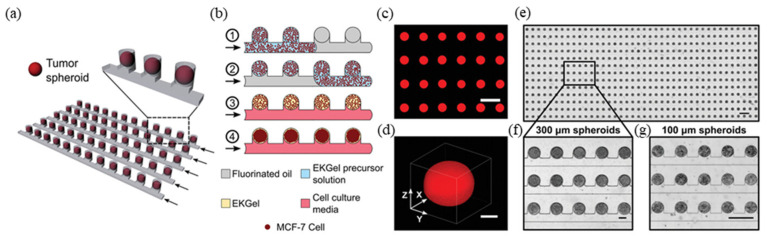
Microfluidic arrays of MCF-7 breast tumor spheroids. (**a**) Schematic of the microwell device with several parallel rows of tumor spheroids. (**b**) Schematic of the microwell-based tumor spheroid generation. (**c**) Fragment of an array of rhodamine-B labelled cell-free microgels, Scale bar is 500 µm. (**d**) 3D confocal microscopy image of the rhodamine-B labelled microgel. The scale bar is 100 µm. (**e**) A portion of an array of 300 µm diameter MCF-7 breast cancer spheroids after 48 h culture. Scale bar is 1 mm. (**f**) Close-up view of array in (**e**). (**g**) 100 µm diameter MCF-7 breast cancer spheroids in an MF array after 48 h of cell culture. The scale bars in (**f**,**g**) are 200 µm. Reprinted with permission from [38].

In the horizontal structure, a drug screening platform in the form of a microarray has also been developed, which implements channels in the form of multiple connected small chambers, enabling the simultaneous cultivation and testing of a few to as many as tens to hundreds of cells (mainly in the form of spheroids) on a single chip. A PDMS-based drug screening platform in the form of a microfluidic array is a large-scale platform that implements 50 cylindrical microwells per row on a 1.2 × 3 cm chip, with a total of 16 such rows, allowing a total of 800 spheroids to be cultured simultaneously [38]. In addition, a concentration gradient formation part was incorporated to allow for large-scale testing of different concentrations (eight different concentrations could be tested at once) (Figure 3). In this study, breast cancer cells were cultured on the constructed microarray chip to screen the efficacy of doxorubicin. This is a major breakthrough in bringing the efficiency of conventional HTS to a microfluidic system that allows for dynamic cultivation. In another study, a relatively small-scale platform was also developed to test different sizes of spheroids at once by forming chambers of different sizes of 300, 500, 700, 900, and 1000 μm [65]. The researchers used the chip to build a blood–brain barrier (BBB) to screen the efficacy of cisplatin and doxorubicin, which are representative anticancer drugs.

**Figure 4:**
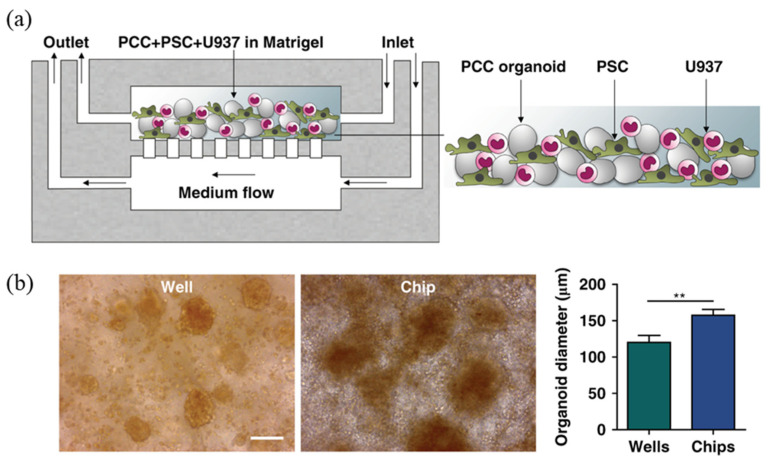
Pancreatic cancer-on-a-chip. (**a**) Schematic of primary cancer cell (PCC)  +  stromal cell seeding on the microfluidic device. (**b**) Comparison between well-grown organoids and chip-grown organoids. Organoids that grew on a chip had a significantly greater diameter than the organoids grown in a welled plate. Scale bar, 100 µm. * *p*  <  0.05, ** *p*  <  0.01. Reprinted from [69] with open access.

**Figure 5:**
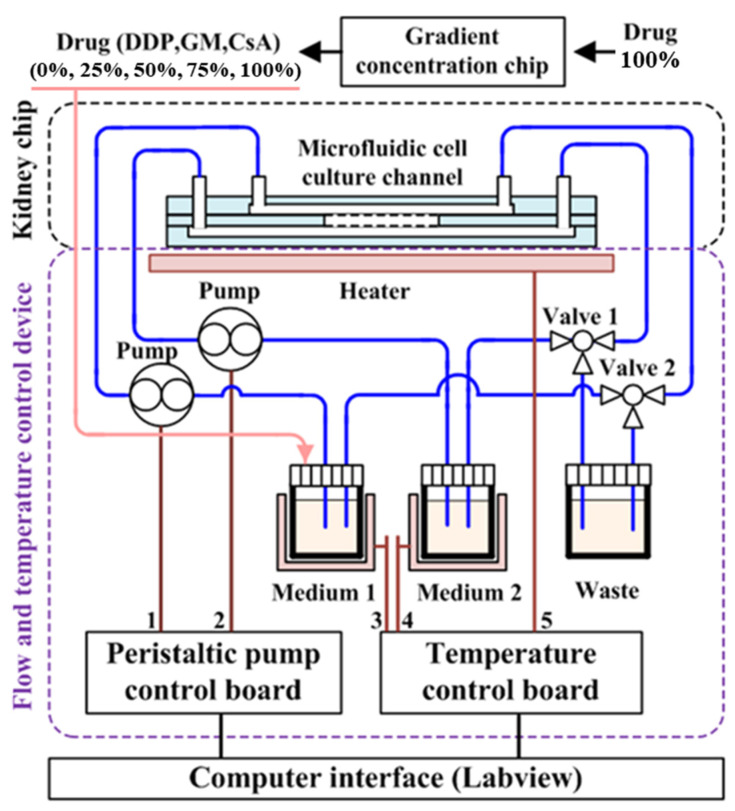
Co-culture microfluidic kidney chip system with flow and temperature control devices. The temperature of the chip and the cell culture medium can be maintained by adjusting the heating plate. Two pumps can regulate the flow rate of the cell culture medium into the chip. Reprinted from [70] with open access.

Vertical structures have the advantage of being more space-efficient, as they can mimic 3D culture structures at a smaller scale Different types of structures can also be fabricated while reducing the size of the chip, and finer flow control is possible when the fluid flow in different layers is controlled independently. In vertical structures, porous membranes are typically adopted and placed between multilayered structures, with cells cultured above or below the membrane attached. The idea is to facilitate interaction through the pores of the porous membrane, a strategy that allows for both effective compartmentalization and interaction. This vertical structure can be seen in a previous study that realized the microenvironment of pancreatic cancer (pancreatic adenocarcinoma) [69], in which the pancreatic microenvironment was realized by compartmentalizing the two chambers with a 0.4 μm pore membrane (Figure 4). The culture chamber at the top was seeded with a mixture of cells and matrigel, and then incubated with media flow at the bottom. The organoids formed in this 3D culture environment grew to a larger scale than in conventional 2D cultures, demonstrating that 3D culture systems are more biomimetic than 2D cultures. The chips were then treated with gemcitabine, ATRA, and Clodrosome, which are used for chemotherapy, either alone or in a cocktail, to verify their efficacy. Based on this vertical structure, another drug screening platform has also been developed with a gradient concentration part and a temperature control part [70]. The researchers implemented an automated system by integrating the LabView interface into a microfluidic system (Figure 5). The researchers used two porous membranes to divide the space inside the chip, making it consist of three layers in total, and attached renal tubular epithelial cells and endothelial cells to each porous membrane to form a PDMS-based kidney chip. The kidney chip was then connected to a gradient concentration chip so that the test drugs (DDP, GM, and CsA) were introduced into the cell chip in a concentration gradient with a total of five concentrations of 0, 25, 50, 75, and 100%. This strategy can maximize the efficiency of drug screening by eliminating the need to manually prepare the concentration of the test drug separately. In addition, a temperature control board was built so that the kidney chip and the culture fluid chamber can be maintained at 37 °C, the optimal cell culture temperature, even if they are not inside an incubator. Although the entire system was not implemented on a single chip, this is a representative study of a drug screening platform that maximizes scalability and efficiency by combining various modules, which is one of the advantages of microfluidic systems.

**Figure 6:**
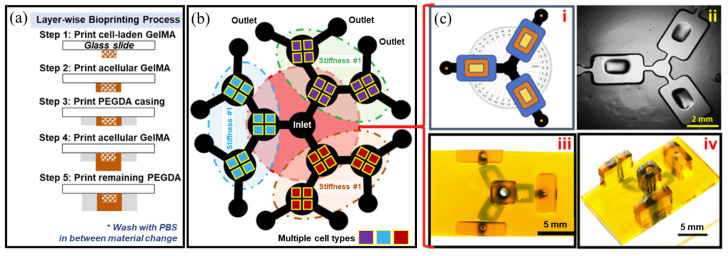
3D bioprinted hydrogel microfluidic chip design. (**a**) Bioprinting process of microfluidic multi-material fabrication. (**b**) The schematic figure of the chip including channels. (**c**) The chosen hydrogel-based microfluidic design; (**i**) The microfluidic chambers arranged in equal angular distribution (120°) for uniform flow rate and discharge with a single inlet and three different outlets, (**ii**) 3D bioprinted preview showing the culture chamber and culture area, (**iii**) top view of the 3D bioprinted microfluidic platform, (**iv**) perspective view of the 3D bioprinted microfluidic platform. Reprinted with permission from [46].

On the other hand, in addition to the various PDMS-based drug screening platforms discussed above, drug screening platforms based on materials such as PMMA or 3D printing have also emerged. PMMA has the advantage of almost no absorption of small molecules as a material for well plates used in conventional 2D platforms, but its application is limited compared to PDMS, which is relatively easy to form fine structures with molds. However, based on the advantages of PMMA, such as relatively low construction cost and mass production, improved drug screening platforms have been developed by incorporating additional structures into PMMA well plates to enable dynamic culture [71]. There were also drug screening platforms built through 3D printing [46]. In this study, 3D structures were created by 3D printing using PEGDA, a representative biocompatible polymer, on a slide glass in the form of the researcher’s intended shape, and GelMA mixed with cells was printed in an adjacent location, showing that a drug screening platform with a relatively complex structure could be built in a short time (Figure 6). In addition, not only the cell culture chamber but also the gradient generator was printed, allowing cells to be treated with different concentrations of the test drug at a time. The efficacy of anti-cancer drug (doxorubicin) was verified in this system. Compared to the traditional cell chip fabrication method, where platform fabrication and cell seeding are separated, this system has almost no time gap between platform fabrication and cell seeding, making it a more efficient and faster cell chip-based drug screening. In addition to these representative examples of cell/organ chip, a more comprehensive set of previous studies can be found in Table 2.

### 3.2. C. elegans Chips for Drug Screening

The *C. elegans* chip-based drug screening platform has a slightly different viewpoint than the cell/organ chips discussed in the previous section. Most of the *C. elegans* chips developed to date have been screened for worm drugs, rather than for substances that can directly improve the symptoms of human diseases or treat them. However, the various microfluidic chips developed in these studies are based on the high genetic homology between *C. elegans* and humans, and may ultimately be utilized as testing platforms for actual human disease-related products in the future, so this review covers these various research achievements in detail. Most of the studies in this part mainly developed PDMS-based microfluidic chips, and some of the studies applied specific materials such as hydrogels to give them special functions [94,95].

When screening for specific substances in *C. elegans* chips, the main parameters analyzed are locomotion [95], including velocity and bending frequency of *C. elegans* swimming; growth rate [96]; life span; and survival rate [50,54,97]. In addition to locomotion, some studies have performed fluorescence imaging-based analyses using mutants that express fluorescence in specific cells (mainly neurons) [40,98], or have analyzed the epigenetic inheritance of certain substances by tracking and observing eggs laid by mothers treated with certain substances, focusing on the advantage of short generation cycles [99]. Other studies have analyzed the effects of certain substances by targeting specific characteristics inherent in *C. elegans* [37].

**Figure 7:**
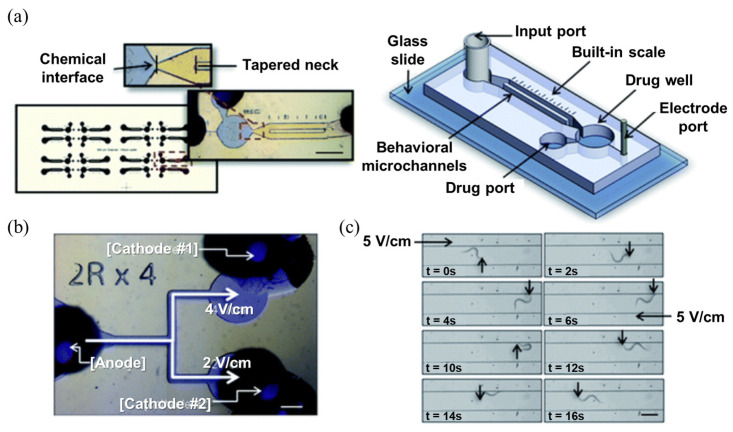
Highly sensitive real-time drug screening platform based on electrotaxis of *C. elegans*. (**a**) Snapshot and schematic figure of the drug screening device. Scale bar = 1.5 mm. (**b**) Snapshot of the fabricated T-shaped microfluidic device showing the three electrode ports and the electric fields of the samples at each port. Scale bar = 750 μm. (**c**) Time-lapse images of a nematode in the straight microchannel. Scale bar = 300 μm. Reprinted with permission from [37].

An intrinsic locomotion characteristic of *C. elegans* that has been utilized for drug screening is “electrotaxis”, a behavioral characteristic that causes *C. elegans* to head toward the cathode under an electric field [100]. This behavioral characteristic is based on neural circuits that are involved in the process of sensing electric fields and moving. Therefore, if the neurons in the relevant neural circuits are damaged by certain substances, the worm may not exhibit normal electrotaxis, or it may show obvious differences, such as a change in the strength of the electric field it responds to, or a slower response time. In contrast, treatment with a substance that has neuroprotective effects may improve the aforementioned symptoms of neuronal damage. Therefore, electrotaxis can be used as a screening target for substances involved in major neurological diseases. In a representative study using electrotaxis as an analytical target, a PDMS-based microfluidic system with input and drug wells on either side of a straight behavioral microchannel was constructed [37]. Electrodes were inserted at both ends of the channel to apply an electric field to the entire channel (Figure 7), and after introducing worms into the chip, an electric field of 5 V/cm was applied to check electrotaxis, and furthermore, levamisole, a type of anthelmintic drug, was selected as a test drug to verify behavioral changes caused by the drug.

Furthermore, one of the key concepts in *C. elegans* chips is immobilization. Since *C. elegans* is a free-moving organism, it is difficult to track and observe specific individuals without providing a confined space, or without a separate tracking method, as they will leave the observation area in a fairly short time. Notably, for the above-mentioned analysis targets, neuronal imaging, it is very challenging to obtain clear enough images from actively moving individuals for quantitative analysis. In this process, traditional studies have either treated the worms with chemicals that cause paralysis or used glue to fix the worms to obtain images [101,102,103,104]. While chemical treatments may seem simple, they can be time-consuming to process and apply, as well as cumbersome to clean up. Another disadvantage of using glue is that the worms cannot be reversibly moved again. Especially in drug screening, the main goal is to evaluate the efficacy and toxicity of different drugs, so the use of different chemicals can affect the accurate drug efficacy/assay [105].

**Figure 8:**
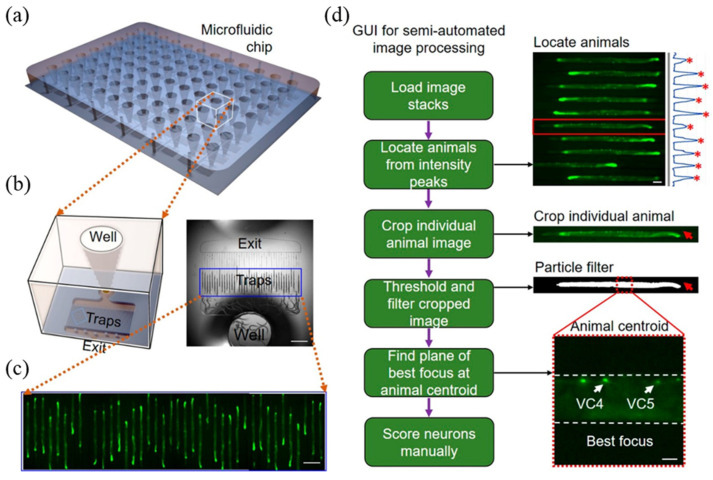
High content screening (HCS) platform for high-resolution imaging and high content analysis of an amyloid precursor protein (APP) model. (**a**) Overall view of the 96-well microfluidic chip for the *C. elegans* HCS platform. (**b**) Close-up illustration (**left**) and image (**right**) of a single well with 40 traps of worms. (**c**) Fluorescence images of a single-copy of the wild-type human amyloid precursor protein (SC_APP) model animals immobilized in 40 traps. (**d**) Schematic of the graphical user interface (GUI), which runs several automated image processing algorithms on the images and then displays the best-focused image of the region of interest, allowing the user to perform rapid phenotypic scoring. Scale bars are 1 mm in (**b**), 200 μm in (**c**), and 100 and 20 μm in (**d**). Reprinted with permission from [29].

Microfluidic chips have shown that these challenges can be overcome with a variety of strategies. For example, a drug screening platform was developed by designing a channel that fits the width of the *C. elegans* body, introducing worms into the channel, and physically immobilizing the worms in a linear shape [29,33]. Based on this strategy, a 96-well microfluidic chip was developed that can be utilized for drug screening [29]. The platform has more than 40 microchannels at the bottom of each well that can trap *C. elegans* in a linear manner, enabling the researchers to test more than 3000 worms in a single 96-well platform with high-throughput (Figure 8). High-quality fluorescence imaging can be effectively achieved with *C. elegans* by using channels designed to match their body width. Additionally, the implementation of semi-automated image processing enables the rapid analysis of these fluorescence images, facilitating the handling of a substantial volume of images efficiently in a short time. After introducing *C. elegans* into the system, *C. elegans* were treated with bexarotene and norbenzomorphan as test drugs, and the degree of neural recovery caused by these substances was analyzed through fluorescence imaging. This HTS-based drug screening platform has been established to screen chemicals that can be indirectly utilized as treatments for human neurodegenerative diseases, with a particular focus on neurodegenerative diseases, and to discover substances that can be utilized as new neurorestorative drugs.

**Figure 9:**
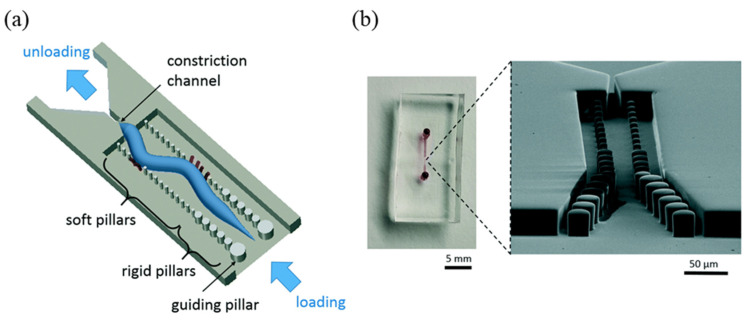
Microfluidic thrashing force assay chip for *C. elegans*. (**a**) Schematic figure of the microfluidic device showing the constriction channel for partial immobilization and the micropillars for lateral force–deflection measurements. (**b**) An optical image (**left**) and scanning electron micrograph (**right**) of the device and channel. Reprinted with permission from [53].

Also, micropillars were effectively used for the immobilization of worms to build a drug screening platform [53]. In this study, two rows of progressively larger diameter pillars were implemented inside the channel (and progressively smaller diameter on the opposite side) with PDMS. By introducing *C. elegans* between the straight channels made of these pillars, the force exerted on the pillars by “thrashing,” the side-to-side movement of the *C. elegans* in the liquid, was measured so that the effects of specific materials could be analyzed (Figure 9). The micropillar implemented inside the channel has soft properties that bend easily with a small force depending on its diameter, while the larger diameter requires a larger force to bend, so the microscopic changes in thrashing force were analyzed in a more multifaceted way. *C. elegans* at various developmental stages were used to quantify the relative changes in thrashing force in response to different glucose concentrations, and further analyzed for efficacy against metformin, a drug used for type 2 diabetes. Further study based on this platform focused the target diseases to neurodegenerative and neuromuscular diseases, and merged thrashing force measurement technology with 2D imaging analysis technology to test a wide variety of drugs in *C. elegans*, demonstrating that a well-implemented platform can be used for ongoing disease-related research [32].

**Figure 10:**
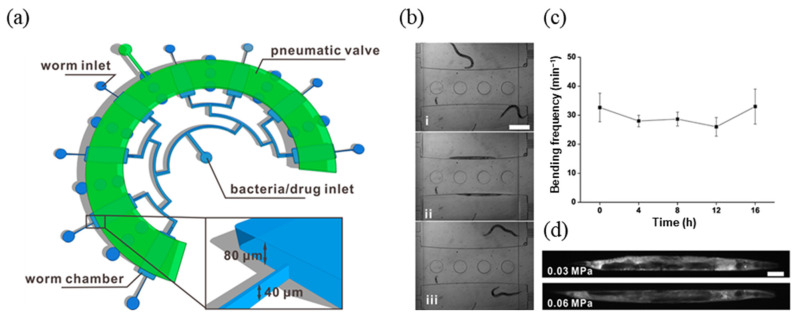
Real-time monitoring microfluidic system for single-animal resolution. (**a**) Schematic figure of the microfluidic device. Close-up view showing the wedge-shaped worm loading channel with 40 µm height. (**b**) Loaded worm shown (**i**) free-moving, (**ii**) immobilized phase, and (**iii**) released phase. (**c**) Bending frequency analysis. Error bar indicates standard error of the mean (SEM), *n* = 4. (**d**) Stress response was not observed in *C. elegans* by analyzing DAF-16 nuclear localization after immobilization for 10 min under the actuator pressure of 0.03 and 0.06 MPa. Scale bar, 50 µm. Reprinted with permission from [98].

Another common technology utilized for system control in *C. elegans* chips is the “pneumatic valve” technology. A pneumatic valve is a method of trapping air in a valve-controlled channel and applying pressure to that channel to open and close separate channels located at the top, bottom, left, and right, utilizing the expansion caused by the compression of the air [106]. Most utilize a thin elastic PDMS membrane, or design the channel walls to be at least as thin as possible during chip fabrication so that when pressure is applied, the elastic PDMS expands and blocks the channel. In *C. elegans* research, elastic PDMS has been utilized for a variety of purposes, including preventing *C. elegans* from moving [98], allowing *C. elegans* to escape during experiments [107], or directly providing mechanical stimuli to *C. elegans* [108]. In drug screening platforms, they are often utilized to obtain precise fluid control and clear images. A study that effectively utilizes pneumatic valves was an antibiotic evaluation platform that utilized a *C. elegans* infection model [98]. In this study, a two-layer microfluidic chip was designed to co-culture *C. elegans* with various pathogenic bacteria while monitoring the immune response for a long period of time to evaluate the effectiveness of antibiotics (Figure 10). The study showed that a pneumatic valve can be used to reversibly immobilize *C. elegans* in the culture chamber. A comparably short time (~1 min) was required to immobilize the *C. elegans* by applying a pressure, obtain fluorescence imaging, and release the *C. elegans*. However, the stress caused to the *C. elegans* by the membrane inflated by the pneumatic valve during this process was verified to be negligible using fluorescent mutants. *P. aeruginosa* infection was then induced, and gentamycin and erythromycin were selected as test drugs to quantitatively analyze the efficacy of these antibiotics.

**Figure 11:**
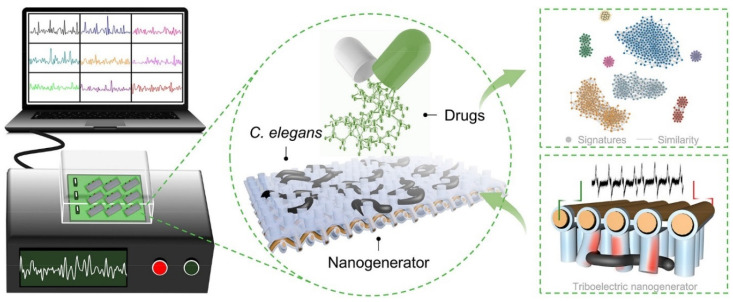
Hierarchically structured biohybrid triboelectric nanogenerators (HB-TENGs) enable high-throughput drug screening on a large scale of *C. elegans*. Reprinted with permission from [30].

Another example is a platform that automatically analyzes the movement of *C. elegans* based on electrical signals [30]. The platform developed in this study was named hierarchically structured biohybrid triboelectric nanogenerators (HB-TENGs), and its core structure is a PDMS-coated copper mesh and a micropillar attached to its bottom. The working principle of the system is based on a combination of electrostatic induction and triboelectric effects. When a *C. elegans* passes between the micropillars, the movement of the *C. elegans* exerts a force on the pillars, which causes the pillars to bend, bringing the copper mesh and PDMS layers into contact, resulting in surface charge transfer and generating an electrical signal. By observing this, the movement of *C. elegans* can be automatically observed and analyzed (Figure 11). Using this platform, a total of 37 different drugs, including epcatechin and apigenin, were treated on *C. elegans* to analyze their effects, and it was confirmed that they exhibited characteristic fingerprints depending on the substance. Therefore, the drug screening platform developed in this study is not only a platform that can analyze the efficacy of specific drugs, but also provides information to quickly verify the effects of various drugs and group drugs with similar results.

As demonstrated in the examples above, microfluidic systems have the advantage of being easily combined with additional devices to maximize efficiency by exploiting the inherent properties of *C. elegans*, as well as allowing a variety of “organismal level” analyses, such as neural circuitry, behavioral responses, and even reproduction. In addition to these representative examples of *C. elegans* chip, a more comprehensive set of previous studies can be found in Table 3.

## 4. Conclusions and Perspectives

The demand for high-throughput and high-resolution platforms in drug discovery continues to grow. In response to the demands, innovative tools called biochips, from cells to model organisms, are being developed that have the potential to change the landscape of drug discovery and high-throughput screening platforms. The development of these technologies has been made possible by the convergence of multiple disciplines, including cell biology, engineering, and medicine.

Addressing the complexity of human physiology remains the most important challenge for the field in the future. While progress has been made in developing biomimetic and model organism assay systems, such as cell/organ chips and *C. elegans* chips, these systems still do not fully reproduce the complex, dynamic, and multi-scale interactions of the living human body. In addition, there is a lack of standardization in the design, fabrication, and operation of cell/organ chip platforms, which can lead to variability in results and hinder reproducibility. Recently, automated systems have been introduced to overcome these limitations, and some are close to commercialization, but paradigm-shifting technologies are not yet fully commercialized. In another respect, while the use of cell/organ chips and *C. elegans* chips can reduce the ethical issues associated with animal testing, they also raise new issues. For example, as organ chip models increase in complexity and realism, there may come a point where these systems themselves become subject to ethical considerations. Of course, these concerns may be premature, but it is a topic we should all be thinking about at this point. Furthermore, while significant progress has been made, many organ chip models still struggle to incorporate components of the vascular and immune systems, which play an important role in a human body’s response to drugs, and research will need to be directed toward filling this gap.

Finally, we would like to conclude by mentioning the recent integration of artificial intelligence (AI) in chip systems. There are studies that have already applied AI to analyze drug screening results [112], and more advanced AI technologies are being applied to drug screening to help revolutionize the drug development process. There are several benefits to integrating AI technology into a drug screening platform. First, AI technology can be used to analyze large amounts of data quickly and accurately to maximize the efficiency of data analysis. In addition to analyzing the resulting data, drug response prediction models can also be built based on AI training data. This allows the efficacy or toxicity of a drug candidate to be predicted in advance. It can also help optimize drug screening parameters and processes to improve the efficiency and accuracy of experiments. Finally, an individual’s genomic and transcriptomic data can be analyzed and used to predict personalized drug response. This could lead to personalized treatment strategies in the future, which could have great synergistic effects.

In this regard, biochip-based drug screening platforms, which have been highly developed over decades, are expected to evolve into higher-level systems in the future, which will actually result in personalized treatment as well as personalized drug screening platforms.

## Figures and Tables

**Table 1:** Characteristics of horizontal and vertical structures.

Chip Sturucture and Examples		Characteristics of Structure	Features	Representative Ref.
Horizontal	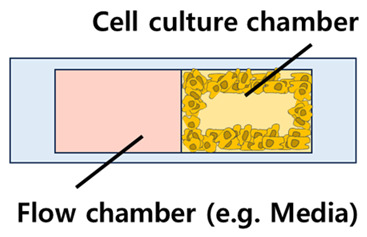	- Mono-layer - Separation method: Post structure (most popular)	- Relatively simple fabrication process - Extra-cellular matrix is required to separate the cells and others effectively	[38,68]
Vertical	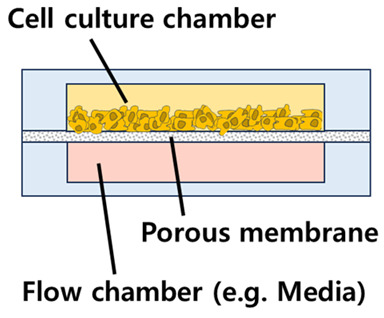	- Multi-layer - Separation method: Porous membrane (most popular)	- Space-efficient, reducing the size of the chip - Finer flow control is possible independently	[69,70]

**Table 2:** Cell/Organ chips for drug screening.

Chip Materials	Cell Line	Test Drugs	Structures	Ref.
PDMS	BT549, T47D	Doxorubicin	Horizontal	[72]
	U-2 OS	Methotrexate	Horizontal	[73]
	MDA-MB-231	Paclitaxel	Horizontal	[68]
		5-fluorouracil, Cisplatin, Docetaxel, Gemcitabine, Irinotecan, Oxaliplatin, Paclitaxel	Vertical	[63]
		Doxorubicin	Horizontal	[74]
	MDA-MB-231, MCF-7, T47D	Doxorubicin, Paclitaxel, Salinomycin, Thiostrepton	Vertical	[75]
	MCF-7	Doxorubicin	Horizontal	[76]
			Horizontal	[38]
		Curcumin, Paclitaxel	Horizontal	[39]
		Cisplatin, Cyclophosphamide, Doxorubicin, Paclitaxel	Vertical	[77]
		Ursolic acid	Vertical	[78]
	THP-1	Cytarabine	Horizontal	[64]
	aPSCs, M2, PANC-1, THP-1,	5-fluorouracil, Gemcitabine, Oxaliplatin, Paclitaxel	Horizontal	[79]
	A431	Doxorubicin	Horizontal	[67]
	A549	Gemcitabine	Horizontal	[80]
		Cisplatin, Doxorubicin	Horizontal	[65]
		Etoposide, Paclitaxel, Vinorelbine	Vertical	[81]
	HT29	5-Fluorouracil	Vertical	[82]
	SW982	Celastrol	Horizontal	[83]
	U251	Docetaxel, Temozolomide	Horizontal	[84]
	U87	Carmustine, Temozolomide	Vertical	[85]
	U937, Human pancreatic stellate cells (PSCs)	ATRA, Clodrosome, Gemcitabine	Vertical	[69]
	Chondrocytes	Resveratrol	Horizontal	[86]
	Renal proximal tubular epithelial cell (RPTEC), peritubular capillary endothelial cells (PCEC)	Cisplatin, Cyclosporin A, Gentamycin	Vertical	[70]
	Primary human prostate cancer cells	Cisplatin	Horizontal	[87]
	Induced hepatic (iHep) cells	Acetaminophen (APAP)	Horizontal	[88]
	Human induced pluripotent stem cell (hiPSC)-derived motoneurons	Riluzole	Horizontal	[89]
	Human stem cell-derived neurons	Clozapine, Clozapine-N-oxide (CNO)	Vertical	[71]
Hydroxyapatite-PDMS	UMR-106	Doxorubicin	Horizontal	[90]
Polystyrene (PS)	HCT116, SW480	Axitinib, Bevacizumab, Sunitinib	Horizontal	[43]
	HUVEC (human umbilical vein endothelial cell)	Bevacizumab, Cetuximab	Horizontal	[91]
Polysulfone	ES bone tumor cell lines	Linsitinib	Horizontal	[19]
PMMA	F9 cell line, HeLa cell line, HeLa-LC3 reporter cell line	Cisplatin	Vertical	[41]
Perfluoro- polyether	NIH3T3	Doxorubicin	Horizontal	[44]
Glass	HTB-37	Amoxicillin, Antipyrine, Digoxin, Ketoprofen	Vertical	[45]
	MDA-MB-231, MCF-10A	Cisplatin, Epirubicin	Horizontal	[92]
Hydrogel	HT-1080	Doxorubicin	Horizontal/ Vertical	[46]
	Patient-derived primary glioblastoma multiforme (GBM) cells	Bevacizumab, Temozolomide	Horizontal	[93]
3D printing resin	A549	5-Fluorouracil, Celecoxib, Cyclophosphamide, Doxorubicin	Vertical	[66]

**Table 3:** *C. elegans* chips for drug screening.

Materials	Strains	Test Drugs	Key Points	Ref.
PDMS	AU133 *agls17(irg-1::gfp),* ERT012 *zip-2(tm4067),* TJ356 *zls356(daf-16::gfp,rol-6)*	Erythromycin, Gentamicin	Long-term monitoring of the immune responses and evaluating the antibiotic effect of antibiotics	[98]
	N2, *glp-4(bn2ts)*, *sek-1(km4)*	Baicalin, Cefepime hydrochloride, Ciprofloxacin, Coptisine, Gypenoside, Meropenem	Automated worm dispensation based micro-sampler	[40]
	N2, SJ4100 *zcIs13[hsp-6::GFP]*	Doxycycline, Tetramisole	High-throughput imaging and analysis for antibiotics test	[109]
	N2	Doxycycline	Observation of the development of life stages during drug testing	[96]
		Hydrogen peroxide	Using the priming valve, the flows were controlled	[55]
		Cu^2+^	Locomotive behavior analysis on neurotoxicity using a micro-injection droplet microfluidic system	[110]
	N2, DA1316 *avr-14(ad1305); avr-15(vu227); glc-1(pk54)* VC2937 *unc-38(ok2896)* CB407 *unc-49(e407)* CB6147 *bus-8(e2882)*	Ivermectin, Levamisole, Piperazine	Pharyngeal pumping was analyzed by microfluidic electropharyngeogram (EPG) to confirm the effect of anthelmintic drugs	[33]
	LS587 *(dys-1(cx18)I; hlh-1(cc561)II),* AM725 *(rmIs290[unc-54p::Hsa-sod-1(127X)::YFP])*, NL5901*([unc54p::alphasynuclein::YFP+ unc-119(+)])*	Doxycycline, Levodopa, Melatonin, Pramipexole, Prednisone, Riluzole	Thrashing and muscle morphology was analyzed in the micropillar platform	[32]
	N2, GZ1326 (expressing mCherry::H2B to mark chromatin and GFP::PH to mark cell membranes)	Cytochalasin-D	A fully integrated microfluidic approach for studies of *C. elegans* early embryogenesis	[31]
	LX959 *vsIs13 [lin-11::pes-10::GFP + lin-15(+)] IV lin-15B(n765) X*, JPS67 *vxSi38 [Prab-3::huAPP695::unc-54UTR, Cb unc-119(+)] II unc-119(ed3) III vsIs13IV*, JPS449 *vxSi38 II; unc119(ed3) III; vsIs13 IV; lin15b(n765), vem-1(gk220) X*, JPS607 *vxSi38 II; unc-119(ed3) III; vxIs13 IV;lin15b(n765) vem-1(ok1058) X*	Bexarotene, Norbenzomorphan	40 trap microchannels at the bottom of each well, enabling the researchers to test more than 3000 worms in a single 96-well platform with high-throughput	[29]
	*hsp-6::gfp*	Doxycycline	Microfluidic platform to observe mother-to-progeny heritable transmission	[99]
	N2, CB4108 *fog-2(q71)V*, AU166 *daf-16(mgDf47) I; fog-2(q71) V*	Hydrogen peroxide, Sodium chloride	The platform with two 50 arena arrays per chip and an imaging capacity of 600 animals per scanning device	[97]
	N2, CL2166 *(dvIs19)*	CdCl_2_	Automated and integrated platform based on *C. elegans* relieving manual operations on worm dispensing, maintenance, imaging, and endpoint analyses	[50]
PDMS-Glass	SJ4100 *(zcIs13[hsp-6p::GFP])*	Doxycycline	Tracked different phenotypic traits of individual *C. elegans* nematodes throughout their full life cycle	[111]
	N2, CF1038 *(daf-16(mu86)I.)*, TJ356 *(daf-16(zIs356)IV)*, CF1553 *(sod-3(muIs84))*, CL2070 *(hsp-16.2(dvIs70))*	Caffeic acid phenethylester (propolis)	The combination use of multiple functional units, including micro-pillar, worm responder, a branching network of distribution channels, and microchambers	[54]
	N2, RB1169 *[oga-1(ok1207)]*, *RB653 [ogt-1(ok430)]*	Metformin	A loss of thrashing force following the introduction of glucose into a wild-type worm culture that could be reversed upon treatment with the type 2 diabetes drug metformin	[53]
PDMS-Copper	N2	Total 37 test drugs including Apigenin, Allomatrine, Baicalin, Epicateching, etc.	In vivo screening strategy combining hierarchically structured biohybrid triboelectric nanogenerators (HB-TENGs) arrays	[30]
PDMS-Hydrogel	N2	Tetramisole	A simple and easy-to-use microfluidic system for automated long-term culturing and phenotyping of *C. elegans* at single-organism resolution	[94]
Hydrogel	N2, CB211 *(lev-1(e211))*, JR667 *(wIs51[SCMp::GFP]; unc-119(e2948))*, OH15089 *(otIs657[klp-6p::mCherry + flp-3p::mCherry + klp-6p::NLG1::spGFP1-10 + flp-3p::NLG1::spGFP11])*	Tetramisole	“Microswimmer combing” rapidly isolated live small animals on an open-surface array	[95]

## Data Availability

The data presented in this study are available on request from the corresponding authors.
